# Genetic variation in *APOL1* and *MYH9* genes is associated with chronic kidney disease among Nigerians

**DOI:** 10.1007/s11255-012-0263-4

**Published:** 2012-09-07

**Authors:** Bamidele O. Tayo, Holly Kramer, Babatunde L. Salako, Omri Gottesman, Colin A. McKenzie, Adesola Ogunniyi, Erwin P. Bottinger, Richard S. Cooper

**Affiliations:** 1 Department of Preventive Medicine and Epidemiology, Loyola University Chicago Stritch School of Medicine, 2160 S. First Ave., Maywood, IL USA; 2 Department of Medicine, University of Ibadan, Ibadan, Nigeria; 3 Charles R. Bronfman Institute for Personalized Medicine, Mount Sinai School of Medicine, New York, NY USA; 4 Tropical Metabolism Research Unit, University of the West Indies, Mona, Jamaica

**Keywords:** Chronic kidney disease, *APOL1*, *MYH9*, Genetic renal disease

## Abstract

**Purpose:**

A region of chromosome 22 which includes *APOL1* and *MYH9* genes was recently identified as a risk locus for non-diabetic forms of kidney disease, including idiopathic and HIV-associated focal segmental glomerular sclerosis and kidney disease clinically attributed to hypertension among African Americans. The purposes of the current study were, therefore, to examine the frequency of these variants and to determine whether they are associated with chronic kidney disease (CKD) among native Africans.

**Methods:**

To investigate the possible evidence of association between variants in these genes and non-diabetic CKD among West Africans, we performed a case/control analysis in a sample of 166 Nigerians without history of European admixture. Our study included a total of 9 variants on *APOL1* (*n* = 4) and *MYH9* (*n* = 5) genes.

**Results:**

We observed significantly strong associations with previously reported *APOL1* variants rs73885319 and rs60910145, and their two-allele “G1” haplotype (*P* < 0.005). We did not observe significant evidence of association between non-diabetic CKD and any of the *MYH9* variants or haplotypes after accounting for multiple testing in our sample.

**Conclusions:**

In conclusion, *APOL1* risk variants are associated with non-diabetic forms of CKD among Nigerians of Yoruba ethnicity. Further information on *APOL1/MYH9* variants may lead to screening programs, which could lead to earlier detection and interventions for non-diabetic kidney disease.

## Introduction

The lifetime risk of end-stage kidney disease (ESKD) among African Americans is threefold higher than that among whites [1]. Differences in access to care and other socioeconomic factors do not entirely account for the markedly higher rates of ESKD among African Americans which suggests a possible role for genetic factors [1–6]. Recently, a region of chromosome 22, which includes *APOL1* and *MYH9* genes, was identified using mapping by admixture disequilibrium as a risk locus for non-diabetic forms of kidney disease, including idiopathic and HIV-associated focal segmental glomerular sclerosis (FSGS) and kidney disease clinically attributed to hypertension [7–9]. Genetic variants in the region show very strong associations with non-diabetic kidney disease. Specifically, a two-allele haplotype termed “G1” consisting of two non-synonymous coding variants rs73885319 (S342G) and rs60910145 (I384M) along with rs71785313—a 6 base pair deletion termed “G2” and close to G1 in exon 5 of *APOL1* [10, 11]—is likely to account for the majority of risk of non-diabetic kidney disease associated with the variants in this region, but the role of *MYH9* variants in non-diabetic kidney disease risk remains controversial [7]. Regardless, variants in the region are more common in individuals of African descent compared with those of European descent [10, 11]. The fact that these variants are absent or less common in other populations has been used to explain the non-replication of these associations in non-African descent populations. The purposes of the current study were, therefore, to examine the frequency of these variants and to determine whether they are associated with CKD among native Africans.

## Subjects and methods

### Study participants and phenotype measurements

The source population for both cases and controls was participants in the Genetics of Hypertension in Blacks study, an ongoing project examining genetic variants for blood pressure among adults from the Yoruba tribe. Participants in the current analyses included 88 and 81 adults with and without non-diabetic kidney disease, respectively. Participants were recruited from the University College Hospital General Medicine and Nephrology clinics, University of Ibadan, Nigeria. The project was reviewed and approved by the Institutional Review Board at the Loyola University Chicago Stritch School of Medicine, Maywood, IL and the Joint Ethical Committee of the University of Ibadan/University College Hospital, Ibadan, Nigeria. The consent process was presented in English or Yoruba, and written informed consent was obtained from all participants.

Cases were defined as individuals of the Yoruba tribe aged 16–70 years with all stages of chronic kidney disease (CKD) of at least 3 months duration who met criteria for non-diabetic CKD clinically associated with long-standing hypertension, or CKD in the presence of proteinuria (Proteinuric CKD) or CKD associated with HIV. Proteinuric CKD was defined as presence of spot urine protein/creatinine ≥2 g/g in the absence of red blood cell (RBC) casts or hematuria on urine microscopy or urinalysis and absence of known causes for CKD such as parasitic or other infections or diabetes. Clinically diagnosed hypertension-associated CKD was based on history or clinical evidence of long-standing hypertension (e.g., electrocardiogram evidence of left ventricular hypertrophy) and a spot urine protein/creatinine ratio <2 g/g, in the absence of other known causes for CKD, consistent with the African American Study of Kidney Disease criteria [12]. CKD associated with HIV was defined as a positive HIV test and absence of other known causes for CKD including diabetes and parasitic diseases. Women who were pregnant, persons with diabetes mellitus, sickle cell disease, hepatitis B or C, acute kidney injury, or other terminal illnesses such as cancer were excluded. Individuals with CKD were recruited by nephrologists at the University College Hospital. Patients were evaluated by physicians in the Nephrology clinic. The clinical exam and laboratory studies included three serial blood pressure measurements using an automated device (Omron HEM-412C) previously evaluated in our field settings [13], anthropometric measures, a complete blood count and metabolic panel, electrocardiogram (ECG), testing for HIV and hepatitis B, urinalysis with microscopy and a bilateral kidney ultrasound when possible. All laboratory analyses in cases were completed at the University College Hospital Laboratory as part of routine clinical workup.

Controls consisted of normotensive individuals (BP < 140/90 in the absence of anti-hypertensive medication use) of the Yoruba tribe without evidence of kidney disease (serum creatinine <1.4 and <1.2 mg/dl in men and women, respectively, and spot urine albumin/creatinine ratio <30 mg/g). All controls had standardized physical exams including three serial blood pressure measurements using the same device as used for the cases; fasting blood samples and a spot urine specimen were also obtained. Serum creatinine was measured in the control specimens at Fairview Laboratory in Minnesota by rate reflectance spectrophotometry using thin film adaptation of the creatinine amidinohydrolase method on the Vitros analyzer (Johnson and Johnson Clinical Diagnostics, Rochester, NY). The laboratory analytic coefficient of variation was 2.2 %.

### Genotyping and quality assessment

Genotyping was carried out on genomic DNA from 88 non-diabetic CKD subjects and 81 non-CKD subjects randomly selected from among the controls described above. The genotyping was performed at the Charles R. Bronfman Institute for Personalized Medicine (Mount Sinai School of Medicine, New York, NY), using a custom Fluidigm™ 96.96 array platform and ABI TaqMan SNP genotyping assays. The assays were originally selected for 96 published disease-associated single nucleotide polymorphisms (SNPs) and included 4 SNPs on *APOL1* and 6 SNPs on *MYH9* genes which have been reported to be associated with kidney disease. The assays also included variants associated with liver disease, type 2 diabetes or drug response. Standard quality control procedures were applied to the genotype data using all SNPs on the chip. As part of the procedures, 3 samples (1 case and 2 controls) and 2 SNPs with proportion of missing genotypes greater than 0.1 were filtered out. We also filtered out SNPs with minor allele frequency less than 0.05 (*n* = 23) or failing Hardy–Weinberg equilibrium test at 0.01 (*n* = 3). The final 68 SNPs that passed quality control included 9 of the 10 *APOL1/MYH9* variants. Since the objective of the current analysis was to examine the association between previously reported kidney disease–associated SNPs and CKD in a sample of Nigerians, subsequent screening for associations with CKD was therefore restricted to just the variants on *APOL1* (rs9622363, rs73885319, rs60910145 and rs71785313) and *MYH9* (rs11912763, rs2032487, rs4821481, rs5750248 and rs5750250) genes.

### Statistical analyses

To test for associations between CKD status and each single SNP, we fitted logistic regression models in which each SNP was presented as a predictor variable whose values were equal to the number of copies of the minor allele (0, 1, 2) (i.e., additive mode), or presence of at least one copy of the minor allele (0, 1) (i.e., dominant mode) or presence of two copies of the minor allele (0, 1) (i.e., recessive mode). The fitted model, which included sex and age as covariates, can be represented as: logit[*pr*(*D* = 1)] = *α* + *β*_1_ *G* + *β*_2_ *sex* + *β*_3_ *age*, where *D* denotes CKD affection status; *G* denotes SNP coded as additive, dominant or recessive; β denotes the corresponding coefficient for each variable in the model (SNP, sex or age), and its exponential is the corresponding odds ratio. To account for multiple comparisons, the statistical significance of each association test was empirically derived by permuting the data set 10,000 times.

To explore the possible associations between CKD status and various joint allelic configurations of multiple SNPs, we performed haplotype association analysis of the *APOL1* and *MYH9* SNPs. We used the software Haploview [14] to compute the estimates of linkage disequilibrium (LD) for each pair of SNPs by the standard D-prime method[15] and to determine the haplotype blocks—regions with no evidence of historical recombination events, but significant level of LD. The haplotype blocks were defined by the four-gamete test [16, 17] as implemented in Haploview. Analysis included all haplotypes with frequencies of at least 10 % within the constructed blocks and also the two-allele haplotypes consisting of rs73885319 and rs60910145 that included G1. All the haplotypes were individually tested for association with CKD by using logistic regression models as described above for tests of genotype association. Age and gender were adjusted for in each haplotype–phenotype analysis.

## Results

After excluding participants whose genotype data did not pass quality control, the study sample consisted of a total of 166 subjects (87 cases and 79 controls). The descriptive characteristics of the cases and controls are presented in Table 1. Among both cases and controls, approximately half were women. On average, the CKD subjects tended to be older (42.1 vs. 35.2 years), heavier (23.2 vs. 21.9 kg/m^2^) and have higher blood pressure (systolic: 136.6 vs. 111.6 mm Hg) when compared with the non-CKD controls. Among the 87 CKD subjects, the physician-reported diagnosis of kidney disease was proteinuric CKD in 35 (40.2 %), HIV nephropathy in 8 (9.2 %) and clinically attributed hypertensive CKD in 44 (50.5 %). All the CKD subjects had a negative hepatitis B surface antigen test within 30 days of enrollment in the study. All but 3 cases had stage 5 CKD (1 with proteinuric CKD and 2 with CKD clinically attributed to hypertension.

**Table 1:** Descriptive characteristics of study subjects

	Non-diabetic CKD cases (*N* = 87)	Controls (*N* = 79)	All (*N* = 166)
No. of females (%)	41 (47 %)	39 (49 %)	80 (48 %)
Age (years)^†^	42.1 ± 16.9	35.2 ± 8.2	38.8 ± 13.9
Body weight (kg)	61.6 ± 11.1	59.8 ± 10.8	60.8 ± 11.0
Height (m)	1.6 ± 0.1	1.7 ± 0.1	1.6 ± 0.1
Body mass index (kg/m^2^)	23.2 ± 4.6	21.9 ± 4.2	22.6 ± 4.4
Systolic blood pressure (mm Hg)^†^	136.6 ± 31.0	111.6 ± 10.0	124.7 ± 26.6
Diastolic blood pressure (mm Hg)^†^	88.1 ± 20.7	66.9 ± 6.1	78.0 ± 18.8

*CKD* chronic kidney disease

Data presented as mean± standard deviation

^†^Values are significantly different (*P* < 0.05) between cases and controls

The frequencies of the risk allele of the 9 *APOL1*/*MYH9* variants among the cases and controls are listed in Table 2. In the current analyses, the coded alleles are the same as the minor alleles of the variants. For any variant with reported odds ratio (OR) greater than 1.0, the coded minor allele is also the risk or disease susceptibility allele. As would be expected, coded risk alleles tended to be more frequent in cases than in controls, for example, 0.442 versus 0.266 and 0.500 versus 0.301 for rs73885319 and rs60910145, respectively. The variant rs71785313, which is an *APOL1* insertion/deletion, had the least frequent minor allele (0.105) in both cases and controls. Results of the covariate-adjusted case/control analyses are also presented in Table 2 for additive, dominant and recessive genetic modes of association. We observed significant associations for two *APOL1* SNPs—rs73885319 and rs60910145 under all three genetic modes of association. Both variants have larger effects under the recessive mode (odds ratios: 3.85 and 3.12 for rs73885319 and rs60910145, respectively) when compared with the additive or dominant mode. The two variants are in almost perfect linkage disequilibrium (D-prime = 1.00, *r* ^2^ = 0.82) and as such, when adjusted for either one, the association for the other disappeared.

**Table 2:** SNP associations with non-diabetic chronic kidney disease in 87 cases and 79 controls

SNP	Gene	Alleles	Coded (minor) allele (frequency, %)	HWE *P* value	Coded allele frequency (%)		Association mode	OR (95 % CI)	*P* value	
					Cases	Controls			Unadjusted	Corrected^‡^
rs9622363	*APOL1*	A/G	A (27.71)	0.788	25.86	29.75	Additive	0.76 (0.45–1.31)	0.326	0.875
							Dominant	0.88 (0.47–1.66)	0.695	0.999
							Recessive	0.24 (0.05–1.29)	0.097	0.377
rs73885319	*APOL1*	A/G	A (35.76)	1.000	44.19	26.58	Additive	2.29 (1.39–3.77)	0.001	0.005
							Dominant	2.59 (1.34–5.00)	0.005	0.025
							Recessive	3.85 (1.31–11.36)	0.015	0.038
rs60910145	*APOL1*	G/T	G (40.61)	0.114	50.00	30.13	Additive	2.04 (1.32–3.17)	0.001	0.006
							Dominant	2.54 (1.31–4.92)	0.006	0.034
							Recessive	3.12 (1.35–7.20)	0.008	0.015
**G2**: rs71785313	*APOL1*	D/I	D (10.54)	1.000	8.62	12.66	Additive	0.61 (0.29–1.31)	0.207	0.701
							Dominant	0.64 (0.29–1.40)	0.263	0.816
							Recessive	NE	NE	NE
rs11912763	*MYH9*	A/G	A (33.13)	1.000	38.51	27.22	Additive	1.68 (1.02–2.76)	0.040	0.197
							Dominant	2.03 (1.06–3.87)	0.032	0.183
							Recessive	1.70 (0.58–4.94)	0.334	0.872
rs2032487	*MYH9*	T/C	T (22.12)	0.770	18.39	26.28	Additive	0.68 (0.40–1.16)	0.157	0.580
							Dominant	0.64 (0.33–1.23)	0.177	0.645
							Recessive	0.55 (0.14–2.22)	0.400	0.934
rs4821481	*MYH9*	T/C	T (22.29)	0.777	18.39	26.58	Additive	0.66 (0.39–1.13)	0.132	0.532
							Dominant	0.61 (0.32–1.18)	0.143	0.583
							Recessive	0.55 (0.14–2.24)	0.407	0.940
rs5750248	*MYH9*	C/T	C (30.72)	1.000	25.86	36.08	Additive	0.61 (0.37–0.99)	0.047	0.225
							Dominant	0.56 (0.29–1.05)	0.071	0.354
							Recessive	0.46 (0.15–1.41)	0.176	0.627
rs5750250	*MYH9*	A/G	A (31.82)	0.635	26.16	37.97	Additive	0.56 (0.34–0.94)	0.027	0.141
							Dominant	0.51 (0.27–0.97)	0.040	0.208
							Recessive	0.44 (0.14–1.38)	0.157	0.576

Adjusted for age and gender

*SNPs* single nucleotide polymorphisms, *HWE* Hardy–Weinberg equilibrium, *OR* odds ratio, *CI* confidence interval, *NE* not estimated

^‡^ *P* values corrected for multiple comparisons

**Fig. 1:**
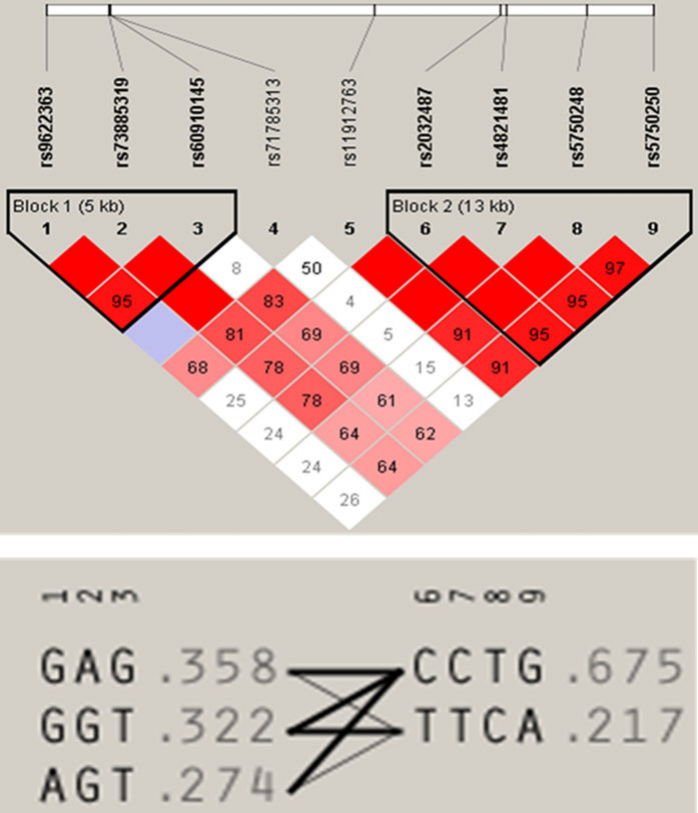
Plot of linkage disequilibrium between SNPs in *APOL1*/*MYH9* region (*top*) and their haplotypes (*bottom*)

The haplotype blocks formed by the *APOL1*/*MYH9* variants are displayed in Fig. 1. There were two blocks consisting of three *APOL1* SNPs and four *MYH9* SNPs, respectively. Two SNPs, rs71785313 on *APOL1* and rs11912763 on *MYH9*, were not included in any of the blocks. The *APOL1* block thus included rs9622363, rs73885319 and rs60910145, and the *MYH9* block included rs2032487, rs4821481, rs5750248 and rs5750250. There were a total of five haplotypes each with a frequency of at least 0.10 (Fig. 1). The results of the covariate-adjusted haplotype associations are presented in Table 3. We also present the results for the two-allele haplotypes of rs73885319 and rs60910145 that included the previously reported G1 haplotype. Haplotype frequencies ranged between 0.217 and 0.675 in the study sample. The association results indicate that the G–A–G haplotype of the *APOL1* SNPs (rs9622363–rs73885319–rs60910145) is a significant risk factor for CKD under any mode of association. The ORs are 2.26 (*p* = 0.005), 2.54 (*p* = 0.023) and 3.79 (*p* = 0.041) for the additive, dominant and recessive modes of association, respectively, after correction for multiple comparisons. Similarly, the two-allele haplotypes of rs73885319 and rs60910145 otherwise termed G1, demonstrated strong levels of association with CKD. The ORs for the G1:rs73885319–rs60910145 (A–G) haplotype are 2.25 (*p* = 0.006), 2.52 (*p* = 0.025) and 3.80 (*p* = 0.041) for the additive, dominant and recessive modes of association, respectively. The crude association [OR, 2.67(95 % CI, 0.79–8.97)] with the compound risk heterozygote state among subjects heterozygous (10 cases and 5 controls) for both the G1:A–G and G2:D risk alleles and subjects (21 cases and 28 controls) without any of the risk alleles was not significant (*P* = 0.143). On the other hand, the G–T haplotype of G1:rs73885319–rs60910145 indicated significant protective association with CKD under the additive (OR = 0.49, *p* = 0.005), dominant (OR = 0.40, *p* = 0.025) and recessive (OR = 0.32, *p* = 0.014) modes. The implication of this is a significantly high CKD risk for those carrying zero copies of the G1:G–T haplotype—with an OR as high as 3.13 (*p* = 0.014). We did not observe significant evidence of association between any of the *MYH9* haplotypes and CKD after accounting for multiple testing (Table 3).

**Table 3:** Haplotype associations with non-diabetic chronic kidney disease in 87 cases and 79 controls

SNP combination	Haplotype	Haplotype frequencies (%)			Association mode	OR (95 % CI)	*P* value	
		Cases	Controls	All			Unadjusted	Corrected^‡^
*APOL1* SNPs								
rs9622363 | rs73885319 | rs60910145	G–A–G	44.25	26.92	35.81	Additive	2.26 (1.37–3.73)	0.001	0.005
					Dominant	2.54 (1.31–4.92)	0.006	0.052
					Recessive	3.79 (1.28–11.20)	0.016	0.024
rs9622363 | rs73885319 | rs60910145	A–G–T	25.29	30.13	27.41	Additive	0.72 (0.42–1.23)	0.231	0.641
					Dominant	0.81 (0.43–1.53)	0.524	0.392
					Recessive	0.24 (0.05–1.27)	0.093	0.983
rs9622363 | rs73885319 | rs60910145	G–G–T	24.71	39.74	32.16	Additive	0.58 (0.37–0.90)	0.015	0.063
					Dominant	0.49 (0.26–0.93)	0.028	0.215
					Recessive	0.41 (0.16–1.03)	0.057	0.134
**G1**: rs73885319 | rs60910145	A–G	44.19	26.92	35.98	Additive	2.25 (1.36–3.71)	0.002	0.005
					Dominant	2.52 (1.30–4.88)	0.006	0.051
					Recessive	3.80 (1.29–11.22)	0.016	0.026
**G1**: rs73885319 | rs60910145	G–T	50.00	69.87	59.45	Additive	0.49 (0.32–0.76)	0.001	0.005
					Dominant	0.32 (0.14–0.73)	0.007	0.018
					Recessive	0.40 (0.21–0.77)	0.006	0.031
*MYH9* SNPs								
rs2032487 | rs4821481 | rs5750248 | rs5750250	T–T–C–A	17.24	26.58	21.67	Additive	0.62 (0.36–1.07)	0.083	0.302
					Dominant	0.57 (0.29–1.09)	0.091	0.899
					Recessive	0.50 (0.11–2.16)	0.352	0.373
rs2032487 | rs4821481 | rs5750248 | rs5750250	C–C–T–G	72.41	62.03	67.45	Additive	1.66 (1.01–2.74)	0.046	0.184
					Dominant	2.16 (0.71–6.60)	0.176	0.299
					Recessive	1.80 (0.95–3.42)	0.073	0.609

Adjusted for age and gender

*OR* odds ratio, *SNPs* single nucleotide polymorphisms, *CI* confidence interval

^‡^ *P* values corrected for multiple comparisons

## Discussion

In this study, we report the findings from a case/control association analysis of non-diabetic chronic kidney disease and variants in *APOL1* and *MYH9* genes in an African sample from southwest Nigeria. *APOL1* and *MYH9* variants are associated with non-diabetic CKD among African Americans [7–9, 11, 18, 19] and Hispanic Americans [20]. The purpose of our study was to investigate the possible evidence of association between these variants and non-diabetic CKD in a sample of Africans of the Yoruba tribe without history of European admixture. We replicated association with *APOL1* gene variants previously reported among African Americans and Hispanic Americans [10, 11]. We observed significant associations with two *APOL1* variants and their haplotypes. The strength of the association between the two-allele haplotype of *APOL1* variants rs60910145 and rs73885319 (G1) and non-diabetic CKD in the current study is about half of the sevenfold-increased odds of hypertensive kidney disease reported among African Americans carrying multiple copies of *APOL1* risk alleles [10]. Stronger associations have been reported with HIV nephropathy [11, 19]. Since sample size only affects statistical significance of estimates and not the strength of the estimates, the apparent attenuation of the observed association when compared to previous findings among African Americans cannot be attributed to sample size. To confirm this, we investigated how much power the current study had to detect significant association between a variant with risk allele frequency at least similar to that observed for G1:A–G (i.e., 0.360), a genetic effect of at least 3.0 under a population risk of 0.00001. Using the software QUANTO[21], we estimated that the current study has at least 80 % power to detect association under recessive mode and over 90 % power under dominant or additive mode.

We noted that the risk allele in the current sample is different for rs73885319 than previously reported [11]. It should be noted that risk allele frequencies for this variant have been observed to differ substantially across African populations [11]. In an African population sample set consisting of 676 samples from 12 African populations that included Cameroon (2 ethnic groups), Congo, Ethiopia (4 ethnic groups), Ghana (2 ethnic groups), Malawi, Mozambique and Sudan, Tzur et al. [11] observed that risk allele frequencies differ between groups from same country and also across the populations. In the Bulsa and Asante populations of Ghana, the frequencies are respectively 0.11 and 0.41, whereas for the Ethiopian groups the frequencies were zero. It is possible that non-diabetic kidney disease risk is not mediated by this variant but rather by other alleles in linkage disequilibrium with the variant or the haplotype G1. As for *MYH9*, we did not find significant association between non-diabetic CKD and any variant or haplotype after correction for multiple testing. A previous study indicated that G1 and G2 are in strong LD with variants in *MYH9* [10], and most of the association previously attributed to *MYH9* variants or haplotypes with CKD is explained by LD with *APOL1* variant rs73885319 [10, 11]. In the present study, we observed reduction in the strength of CKD association with *MYH9* variants when we accounted for the *APOL1* variants by conditioning on either rs73885319 or rs60910145 in the regression models fitted for each of the *MYH9* variants. We note that the observed low LD (*r* ^2^ ≤ 0.12) between *APOL1* and *MYH9* variants in our study sample may have contributed to the observed non-significant association of *MYH9* variants with CKD.

Among African Americans, the frequency of the *APOL1* risk allele is around 0.33 with the high-risk genotype frequency being about 0.11 [22]. Previous studies have reported strong associations with *APOL1* risk alleles G1 (rs73885319 and rs60910145) and G2 (rs71785313) in an autosomal recessive fashion. Inheritance of 2 *APOL1* risk alleles (G1 and G1, G1 and G2, or G2 and G2) increases the risk of non-diabetic kidney disease by over sevenfold. For HIV nephropathy, associations may exceed 30-fold [22]. In this study, we noted a fairly strong association between the G1 risk allele in a recessive model but no association was noted between G2 and non-diabetic kidney disease. This is likely due, in part, to the effect of sample size and the observed low minor allele frequency for G2. It is also possible that differences in the association between *APOL1* variants and CKD in this study could, in part, be due to misclassification of cases or gene × environment interactions but these interpretations remain speculative.

In this study, the strength of the associations between 2 SNPs included in the *MYH9* E1 risk haplotype and non-diabetic kidney disease, though not significant after adjusting for multiple comparisons, was similar to associations reported in cases with end-stage kidney disease clinically attributed to hypertension [18]. The *MHY9* E1 risk haplotype consists of 4 SNPs (rs4821480, rs2032487, rs4821481 and rs3752462); robust associations have been documented in several studies between the E1 haplotype and non-diabetic kidney disease including idiopathic and HIV-associated FSGS and CKD clinically attributed to hypertension [8, 9, 18, 20]. *APOL1* variants are in strong linkage disequilibrium with *MYH9* variants [7], and it remains controversial whether *MYH9* variants play a direct role in kidney disease risk [23, 24].

Due to limited resources and poor access to healthcare for non-urban areas of Nigeria, many patients who present for diagnostic workup and treatment of kidney disease do not undergo kidney biopsy in Ibadan. Diagnosis is typically based on patient’s history, physical exam and laboratory testing. Misclassification of kidney disease is likely in this sample. However, diabetes prevalence is very low in Nigeria [25, 26], and patients with evidence of diabetic kidney disease as determined by the treating physician were excluded from participation in study. The small sample size limited the ability to determine associations with specific types of CKD such as hypertensive CKD or focal segmental glomerulosclerosis. However, even with the small sample, fairly strong associations were noted with *APOL1* variants and the haplotype G1. The associations between *APOL1* variants and non-diabetic CKD among Nigerians of the Yoruba tribe demonstrate that the impact of these genetic factors on CKD risk appear to be independent of the environment. Diet, lifestyle and social structure are dramatically different in Nigeria compared to the United States and other industrialized countries. Hypertension, diabetes and obesity prevalence are markedly lower in Nigeria compared to the African American population [25–29]. Despite these differences, *APOL1* variants are associated with non-diabetic CKD in this population.

The discovery of the *APOL1/MYH9* chromosomal 22 region as a region harboring genetic variants for non-diabetic CKD risk may be very important. It is possible that further delineation of the role of *MYH9* and *APOL1* variants may lead, in the future, to improved screening programs, prevention strategies and clinical interventions for CKD, one of the most common end-organ causes of morbidity and mortality worldwide. The perceived public health importance of these genetic variants is demonstrated by a patent application by the National Institutes of Health for these variants [30]. This study demonstrates that these variants are also operative for non-diabetic kidney disease risk among Nigerians of the Yoruba tribe. Data suggest that the G1 and G2 variants in the *APOL1* region emerged in this population several thousand years ago as a result of conferring protection from *Trypanosoma brucei rhodesiense* [10, 31], a story very similar to the rise in frequency of the sickle cell trait as a result of resistance to certain forms of malaria [32].

## Conclusions

In conclusion, *APOL1* risk variants are associated with non-diabetic forms of CKD among Nigerians of Yoruba ethnicity. Further information on *APOL1/MYH9* variants may lead to screening programs which could lead to earlier detection and interventions for non-diabetic kidney disease.
